# Diet restriction in Ramadan and the effect of fasting on glucose levels in pregnancy

**DOI:** 10.1186/1756-0500-7-392

**Published:** 2014-06-24

**Authors:** Latifa Mohammad Baynouna Al Ketbi, Nico JD Niglekerke, Sanna M Zein Al Deen, Hisham Mirghani

**Affiliations:** 1 Abu Dhabi Health Services, SEHA, Ambulatory Health Care Services, Al Ain, UAE; 2 Community Medicine Department, Faculty of Medicine and Health Sciences, Al Ain, UAE; 3 Obstetrics and Gynecology Department, Faculty of Medicine and Health Sciences, Al Ain, UAE

## Abstract

**Background:**

Maternal diet restriction might be associated with adverse maternal and perinatal outcomes due to metabolic changes. This study aimed to investigate the prevalence of changes in glucose levels due to Ramadan fasting in Emirati pregnant women. We conducted a cross-sectional observational study of 150 women from the United Arab Emirates, (76 during Ramadan and 74 after Ramadan), with uncomplicated pregnancies at a gestational age between 20 and 36 weeks.

**Results:**

The two groups of pregnant women had similar physiological parameters. Using the oral glucose tolerance test, the mean random blood glucose level after 1 hour of breaking the fast was significantly higher (p = 0.002) in the Ramadan fasting group than in the control group, and this was not affected by the number of fasting days. In 50% of patients after Ramadan and 70.5% during Ramadan, this value was more than 6.7 mmol/l, which is high and not an acceptable postprandial level in pregnancy.

**Conclusion:**

Caregivers need to consider the 1-hour postprandial glucose level response after fasting in Muslim pregnant women. Research of an interventional design is required to determine remedial actions for this issue.

## Background

Maternal diet restriction might be associated with adverse maternal and perinatal outcomes. Prentice *et al*[1] reported a higher rate of fetal loss and low birth weight, and increased frequency of neonatal admission to the special care baby unit in women observing Ramadan. Maternal diet restriction is associated with an increased maternal risk of gestational diabetes mellitus and induction of labor [2,3], The reason for this association with an adverse outcome is not completely clear.

Herrmann *et al*[3] suggested that the associated physiological stress that results from diet restriction is associated with increased levels of corticotrophin-releasing factor, epinephrine, norepinephrine, and insulin secretion. In addition, levels of placental steroids and peptide hormones, such as progesterone and estrogens, linearly rise throughout the second and third trimesters, and this results in increased tissue insulin resistance. Therefore, insulin levels in the late third trimester are 50% higher than those in non-pregnant women. Consequently, glucose levels are lower in pregnant women because of hyperplasia of the insulin-secreting pancreatic beta cells, increased insulin secretion, and an early increase in insulin sensitivity followed by progressive insulin resistance. Additionally, increased storage of tissue glycogen, increased peripheral glucose use, decreased hepatic glucose production, and glucose consumption by the fetus (especially in late pregnancy) contribute to approximately 10% to 20% lower glucose levels during pregnancy.

The above-mentioned pathophysiology is an important metabolic adaptation to ensure that the fetus has a good supply of fuel and nutrients at all times through the switch from carbohydrate to fat use. This switch is facilitated by induced insulin resistance and increased plasma concentrations of lipolytic hormones. However, this results in large fluctuations in glucose and insulin levels (fed and fast states). The fasted state is where alternative fuels are made available to the mother, while glucose is reserved for the fetus, and insulin levels are higher in the fasting and postprandial states. After an overnight fast, the maternal fasting capillary whole blood glucose concentration falls, while plasma ketone and free fatty acid concentrations rise.

If another metabolic challenge is added as diet restriction or fasting during the holy month of Ramadan, insulin resistance is aggravated. This phenomenon is described as “accelerated starvation” during pregnancy [4,5]. We hypothesize that in the presence of genetic predisposition, as in the United Arab Emirates (UAE) population, this may increase the metabolic challenge and gestational diabetes mellitus may occur when a woman’s pancreatic function is not sufficient to overcome the insulin resistance.

During the month of Ramadan, healthy adults abstain from food and drink from dawn to sunset. Pregnant women are allowed, if they choose, to postpone the Ramadan fast until after delivery. However, most of the women would like to fast with their families rather than doing this alone later. Therefore, most Muslim pregnant women fast during the holy month of Ramadan. Consequently, diet restriction and fasting could adversely affect pregnancy outcome, especially if Ramadan falls during the hot summer months with long days of fasting.

This situation was clearly described in the recent American Diabetic Association Guidelines [6], which recommend that women with pre-gestational or gestational diabetes are at high risk and are strongly advised not to fast during Ramadan. Nevertheless, this recommendation is not based on any research in this area.

The prevalence of gestational diabetes mellitus is on the rise worldwide. In the UAE population, there is a high prevalence of type-2 diabetes, as well as other disorders associated with metabolic syndrome, such as obesity and dyslipidemia. Therefore, fasting may disproportionately affect fetal and maternal health. Strong empirical evidence of this effect was provided by the HAPO study [7,8] which identified strong continuous associations between several adverse perinatal outcomes and maternal glucose levels, even for glucose levels below those diagnostic of diabetes.

This study aimed to investigate the effect of maternal diet restriction on glucose levels and glycemic control during normal pregnancy. We quantified if the effect size, if any, is significant in relation to guide-related recommendations.

## Methods

We performed a cross-sectional observational study. Patients were recruited from three primary health centers, including Al Yahar, Al Maqam, and Al Khabisi PHC, in Al Ain District, Abu Dhabi, UAE. Inclusion criteria were UAE nationals, uncomplicated pregnancies, gestational age between 20 and 36 weeks, normal oral glucose tolerance test (OGTT) blood glucose levels, and an expressed will to fast during Ramadan. Patients with known medical conditions, multi-fetal pregnancies, and fetal anomalies were excluded. For each fasting mother, a non-fasting healthy pregnant woman matched for age, parity, and gestational age was recruited after the fasting month of Ramadan as a control. Pregnant women were provided with glucometers, and taught how to perform measurements at home by a trained nurse. They were asked to measure their blood glucose level just before breaking their fast and 1 hour postprandial. Pregnant women were requested to adhere to their usual diet during the day of surveillance and to limit variability of food intake. A food sheet was provided to each woman with suggestions of possible food that may be taken after breaking the fast. Recruitment started from the first day of the month of Ramadan until the 30th day. All of the women were fasting continuously until the day of the surveillance. The average hours of fasting were 14 hours during the months of August and September 2010. Written informed consent for participation in the study was obtained from all of the participants. The study was approved by the Al Ain Medical District Human Research Ethics Committee.

A dedicated nurse collected patients’ information and explained the study procedure and followed patients afterwards. A sample size calculation indicated the need for 64 subjects in each group with a power of 80%.

A total of 150 pregnant women were recruited for the study. Seventy-six were recruited during the month of Ramadan (i.e., they were exposed to at least 14 hours of diet restriction) and 74 were recruited during normal months (controls) with no diet restriction. During Ramadan, we measured glucose levels for the 76 women during fasting and at 1 hour postprandial. In the 74 control women who were not studied during Ramadan, we measured fasting blood glucose levels early in the morning, after bed, and at 1 hour postprandial after their main meal.

Collected data were analyzed using SPSS version 19. Standard statistical methods, such as the Pearson chi-square test for analysis of differences in categorical outcomes between the two groups, were used. A p value of < 0.05 was considered to be significant.

## Results

**Figure 1:**
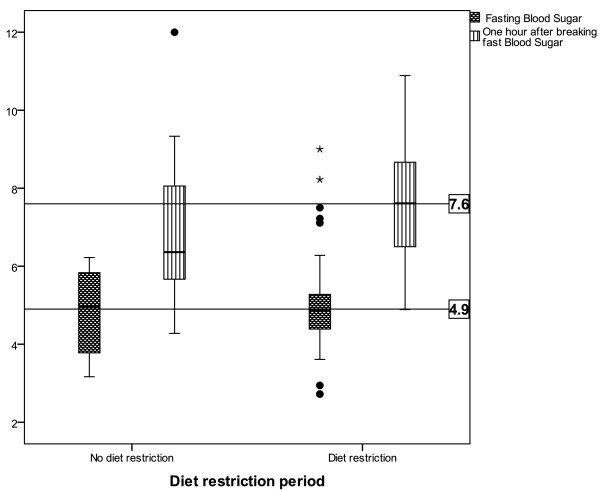
Fasting blood glucose and blood glucose levels after 1 hour of breaking fasting.

The two groups of pregnant women had similar physiological parameters. There were no significant differences in maternal age, parity, body mass index, and gestational age at recruitment between the two groups (Table 1). The mean random blood glucose level after 1 hour of breaking the fast was significantly higher in the Ramadan group who were exposed to long hours of fasting than in the control group (p = 0.02) (Figure 1), but there was no significant difference in glucose levels during the OGTT between the two groups (Table 2). The above-mentioned findings were not affected by the number of fasting days.

**Table 1:** Characteristics of the patients with no diet restriction and with diet restriction

**Descriptive statistics**				
**Ramadan**		**N**	**Mean**	**Std. deviation**
No diet restriction	Gestational age	74	27.0	3.3
	Gravida	74	4.2	3.1
	Parity	72	3.0	2.8
	Age	72	27.5	6.4
	First trimester FBS	55	4.7	.66
	First trimester I hour after breakfast	9	5.0	1.01
	Fasting g OGTT	61	4.5	1.1
	At 1 hr OGTT	51	8.0	2.28
	At 2 hr OGTT	58	6.5	1.7
	Systolic blood pressure	56	103.8	10.05
	Diastolic blood pressure	56	63.3	8.9
	BMI	71	28.6	5.6
	Patient recorded fasting blood sugar	74	5.1	1.0
	Patient recorded post prandial blood sugar	74	6.8	1.5
Diet restriction period	Gestational age	75	27.9	2.9
	Gravida	75	3.7	2.3
	Parity	64	2.5	2.1
	Age	75	28.4	5.4
	First trimester FBS	38	4.6	.56
	First trimester I hour after breakfast	14	5.3	.93
	Fasting g OGTT	52	4.7	.64
	At 1 hr OGTT	43	7.6	2.1
	At 2 hr OGTT	44	6.2	1.4
	Systolic blood pressure	36	105.8	14.0
	Diastolic blood pressure	36	62.1	9.8
	BMI	75	28.7	5.2
	Patient recorded fasting blood sugar	76	4.9	1.0
	Patient recorded post prandial blood sugar	76	7.7	1.4

No significant differences were found in any of the characteristics studied between the two groups. BMI body mass index, OGTT oral glucose tolerance test.

**Table 2:** Differences in recorded blood glucose levels between the two groups

	**Diet restriction period**			**No diet restriction**			**P value**
	**N**	**Mean**	**Std. deviation**	**N**	**Mean**	**Std. deviation**	
Patient recorded fasting blood sugar	76	4.9	1.0	74	5.1	1.0	>0.05
Patient recorded post prandial blood sugar	76	7.7	1.4	74	6.8	1.5	<0.02

In 50% of patients after Ramadan and 70.5% during Ramadan, glucose levels were higher than 6.7 mmol/l (Table 3).

**Table 3:** Patients with glucose levels less than or more than 6.7 mmol/l (acceptable cut point of normal postprandial) in the two groups

**No diet restriction % (no.)**	**Diet restriction % (no.)**	**Blood glucose level**
50 (37)	28.9 (22)	<6.7
50 (37)	71.1 (54)	> = 6.7

## Discussion

In the current study, we observed significantly higher 1-hour postprandial glucose levels in fasting pregnant women compared with the control group. One hour after breaking the fast in women without diabetes or gestational diabetes, according to their OGTT, more than two thirds of them (71.1%) had glucose levels that generally exceeded recommended values [9], which should not exceed 105 mg/dl in normal pregnancies [10]. Whether this finding is unique to this population is unknown because there are no similar studies. Although recent American Diabetic Association guidelines suggest that fasting adversely affects the outcome of pregnancy, which is not based on any previous research [6], Kiziltan *et al*[11] observed no significant changes in fasting glucose levels in pregnant women fasting for Ramadan. However, changes in postprandial glucose levels, when levels are expected to be highest, were not investigated. Interestingly, Mirghani *et al*[12,13] observed a significant decline in fasting blood glucose levels compared with the control group. This finding is in contrast to our study, which showed no significant difference in fasting glucose levels between the two groups. However, the long-term effects of these transient high blood glucose levels, if any, are unclear. Specifically, it is unclear whether an adverse pregnancy outcome in women with high blood glucose levels also occurs in pregnant women who fast during Ramadan. If this is the case, Ramadan fasting, which involves long hours without food or drink and is often followed by excessive meals when the fast is broken, may result in a poor outcome of pregnancy. This could have implications for maternal health care services when interpreting postprandial results of pregnant women who are on a restricted diet.

Previous studies have shown a wide and profound range of biochemical changes associated with maternal fasting. These changes include a significant fall in glucose, insulin, lactate, and creatinine levels, and a rise in triglyceride, non-esterified fatty acid, and 3-hydroxybutyrate levels [2]. Prentice *et al*[1] related the metabolic changes observed in pregnant women who fast during Ramadan to an accelerated starvation phenomenon. Mirghani and colleagues [12] found no effect of fasting on umbilical artery flow, but reported that it significantly affected fetal breathing movements. However, these studies had small sample sizes and were performed in different ethnic groups than our study. In addition, none of the studies studied glucose levels after breaking the fast. The abnormal glucose levels found in the current study may be due to disturbed glucose homeostasis as a result of fasting-related factors. A number of changes in Ramadan may increase stress because of changes in the quality and quantity of the diet, changes in sleeping hours, changes in meal frequency and timing, and the long hours of starvation. The adverse impact of these factors may be aggravated in women who are vulnerable because of glucose intolerance, gestational diabetes, or diabetic patients.

The effect of Ramadan fasting has been investigated in healthy volunteers and in some chronic diseases, such as chronic kidney disease. In healthy volunteers [14-17] a favorable effect of Ramadan fasting was found on major metabolic parameters, such as weight and body mass index. However, conflicting effects were found for lipid profiles, where some studies found a favorable effect of Ramadan fasting on lipid profiles, and other studies found a worsening of lipid profiles. In diabetes, glycemic indicators were found to improve or not change with Ramadan fasting, while in chronic kidney disease, renal function was not affected, except in pre-dialysis cases [18-21].

Variability of diet intake could be a factor in the differences between the two groups of patients (Ramadan and after Ramadan). This is because there is only one main meal in Ramadan following the 14 hours of fasting compared with three meals distributed over long days during non-Ramadan times. However, we believe that variability of diet intake did not significantly influence our findings because people do not tend to eat at levels that induce pathological hyperglycemia. In addition, similar dietary recommendations were given to both groups. Nevertheless, regardless of the underlying cause, internally, externally, or both, hyperglycemia may affect the mother and fetus.

None of the patients performed the OGTT during Ramadan because it would have conflicted with religious obligations. Therefore, self-measurements at home were only a reflection of glucose levels. Additionally, it was the only acceptable to answer the study question on a community based population recruitment. Agarwal [22] found that the portable, plasma optimized glucometer is a good screening test for gestational diabetes with a good correlation with venous samples. Although recent guidelines suggest that only venous blood samples should be used for diagnostic purposes, the use of home monitoring for glucose levels is well established. Home monitoring of glucose values might be appropriate because the glucose values reflect the patients’ life, while tests performed in the hospital, except for fasting blood glucose, reflect one reading, which is variable according to the subjects’ diet and activity. Could the later explains why the high home monitoring postprandial levels in both groups not going with the normal glucose levels in pregnancy not to exceed 6.7 mmol/l. In our sample, 50% of the patients after Ramadan and 71.1% during Ramadan had glucose values higher than 6.7 mmol/l/. Raising a possibility that home monitoring does not correlate with OGTT diagnostic levels which is an important future research area. Another research area that needs to be addressed is the effect of glucose values on the mother and fetus with regard to symptoms and fetal monitoring. In addition, interventional studies need to be performed to enable fasting guidelines, especially in diabetic and gestational diabetic patients.

To maintain normal post-meal glucose levels, pregnant women should be advised to skip meals or those fasting for Ramadan could start eating gradually with healthy low caloric food. Unfortunately, randomization for fasting was not possible in the current study because fasting is based on patients’ religious beliefs. However, to the best of our knowledge, this is the first community-based study report on glucose levels measured at home in fasting pregnant women. Further research is needed to investigate the effect of changes in glucose levels during fasting on the mother and fetus.

## Conclusions

Our study shows that 1-hour postprandial glucose levels are significantly higher in Ramadan fasting pregnant women than in non-fasting women. The main recommendation based on this study is to alert caregivers of Muslim pregnant women to postprandial responses to fasting. In addition, interventional research to determine remedial actions is required.

## Competing interests

The authors declare that they have no competing interests.

## Authors’ contributions

LMB conceptualized, conducted, and wrote the manuscript. NJDN performed statistical analysis and reviewed the manuscript. SMZ conducted the study. HM conceptualized the study and reviewed the manuscript. All authors read and approved the final manuscript. All authors read and approved the final manuscript.

## References

[B1] Prentice AM Prentice A Lamb WH Lunn PG Austin S Metabolic consequences of fasting during Ramadan in pregnant and lactating women Hum Nutr Clin Nutr 1983 37 283 294 6643131

[B2] Mirghani HM Hamud OA The effect of maternal diet restriction on pregnancy outcome Am J Perinatol 2006 23 21 24 16450268 10.1055/s-2005-923435

[B3] Herrmann TS Siega-Riz AM Hobel CJ Aurora C Dunkel-Schetter C Prolonged periods without food intake during pregnancy increase risk for elevated maternal corticotropin-releasing hormone concentrations Am J Obstet Gynecol 2001 185 403 412 11518900 10.1067/mob.2001.115863

[B4] Malhotra A Scott PH Scott J Gee H Wharton BA Metabolic changes in Asian Muslim pregnant mothers observing the Ramadan fast in Britain Br J Nutr 1989 61 663 672 2667640 10.1079/bjn19890153

[B5] Metzger BE Ravnikar V Vileisis RA Freinkel N “Accelerated starvation” and the skipped breakfast in late normal pregnancy Lancet 1982 1 588 592 6121184 10.1016/s0140-6736(82)91750-0

[B6] Al-Arouj M Assaad-Khalil S Buse J Fahdil I Fahmy M Hafez S Hassanein M Ibrahim MA Kendall D Kishawi S Al-Madani A Nakhi AB Tayeb K Thomas A Recommendations for management of diabetes during Ramadan: update 2010 Diabetes Care 2010 33 1895 1902 20668157 10.2337/dc10-0896 PMC2909082

[B7] Hyperglycemia and Adverse Pregnancy Outcome (HAPO) Study: associations with neonatal anthropometrics Diabetes 2009 58 453 459 19011170 10.2337/db08-1112 PMC2628620

[B8] YogevChenHod Coustan Oats McIntyre Metzger Lowe Dyer Dooley Trimble McCance Hadden Persson Rogers Hyperglycemia and Adverse Pregnancy Outcome (HAPO) Study Cooperative Research Group Hyperglycemia and Adverse Pregnancy Outcome (HAPO) study: preeclampsia Am J Obstet Gynecol 2010 202 255.e1 255.e7 20207245 10.1016/j.ajog.2010.01.024 PMC2836485

[B9] Yogev Y Ben-Haroush A Chen R Rosenn B Hod M Langer O Diurnal glycemic profile in obese and normal weight nondiabetic pregnant women Am J Obstet Gynecol 2004 191 949 953 15467570 10.1016/j.ajog.2004.06.059

[B10] Cioni R Carignani L Mignosa M La Torre P Mello G Third-trimester maternal glucose levels from diurnal profiles in nondiabetic pregnancies: correlation with sonographic parameters of fetal growth Diabetes Care 2001 24 1319 1323 11473063 10.2337/diacare.24.8.1319

[B11] Kiziltan G Karabudak E Tuncay G Avsar F Tuncay P Mungan O Meral P Dietary intake and nutritional status of Turkish pregnant women during Ramadan Saudi Med J 2005 26 1782 1787 16311666

[B12] Mirghani HM Weerasinghe S Al-Awar S Abdulla L Ezimokhai M The effect of intermittent maternal fasting on computerized fetal heart tracing J Perinatol 2005 25 90 92 15526011 10.1038/sj.jp.7211221

[B13] Mirghani HM Weerasinghe SD Smith JR Ezimokhai M The effect of intermittent maternal fasting on human fetal breathing movements J Obstet Gynaecol 2004 24 635 637 16147601 10.1080/01443610400007844

[B14] Unalacak M Kara IH Baltaci D Erdem O Bucaktepe PG Effects of Ramadan fasting on biochemical and hematological parameters and cytokines in healthy and obese individuals Metab Syndr Relat Disord 2011 9 157 161 21235381 10.1089/met.2010.0084

[B15] Salehi M Neghab M Effects of fasting and a medium calorie balanced diet during the holy month Ramadan on weight, BMI and some blood parameters of overweight males Pak J Biol Sci 2007 10 968 971 19069900 10.3923/pjbs.2007.968.971

[B16] Lamine F Bouguerra R Jabrane J Marrakchi Z Ben Rayana MC Ben Slama C Gaigi S Food intake and high density lipoprotein cholesterol levels changes during ramadan fasting in healthy young subjects Tunis Med 2006 84 647 650 17193859

[B17] Fakhrzadeh H Larijani B Sanjari M Baradar-Jalili R Amini MR Effect of Ramadan fasting on clinical and biochemical parameters in healthy adults Ann Saudi Med 2003 23 223 226 16985327 10.5144/0256-4947.2003.223

[B18] Shariatpanahi ZV Shariatpanahi MV Shahbazi S Hossaini A Abadi A Effect of Ramadan fasting on some indices of insulin resistance and components of the metabolic syndrome in healthy male adults Br J Nutr 2008 100 147 151 18053308 10.1017/S000711450787231X

[B19] Bouguerra R Belkadhi A Jabrane J Hamzaoui J Maâtki C Ben Rayana MC Ben Slama C Metabolic effects of the month of Ramadan fasting on type 2 diabetes East Mediterr Health J 2003 9 1099 1108 16450543

[B20] Khatib FA Shafagoj YA Metabolic alterations as a result of Ramadan fasting in non-insulin-dependent diabetes mellitus patients in relation to food intake Saudi Med J 2004 25 1858 1863 15711655

[B21] Ziaee V Razaei M Ahmadinejad Z Shaikh H Yousefi R Yarmohammadi L Bozorgi F Behjati MJ The changes of metabolic profile and weight during Ramadan fasting Singapore Med J 2006 47 409 414 16645692

[B22] Agarwal MM Dhatt GS Safraou MF Gestational diabetes: using a portable glucometer to simplify the approach to screening Gynecol Obstet Invest 2008 66 178 183 18562798 10.1159/000140602

